# Inhibition of Three Potato Pathogens by Phenazine-Producing *Pseudomonas* spp. Is Associated with Multiple Biocontrol-Related Traits

**DOI:** 10.1128/mSphere.00427-21

**Published:** 2021-06-02

**Authors:** Adrien Biessy, Amy Novinscak, Renée St-Onge, Geneviève Léger, Antoine Zboralski, Martin Filion

**Affiliations:** a Department of Biology, Université de Moncton, Moncton, New Brunswick, Canada; b Saint-Jean-sur-Richelieu Research and Development Centre, Agriculture and Agri-Food Canada, Saint-Jean-sur-Richelieu, Quebec, Canada; University of Wisconsin—Madison

**Keywords:** *Pseudomonas*, phenazine, *Solanum tuberosum*, biocontrol, *Streptomyces scabies*, *Phytophthora infestans*, *Verticillium dahliae*

## Abstract

Phenazine-producing Pseudomonas spp. are effective biocontrol agents that aggressively colonize the rhizosphere and suppress numerous plant diseases. In this study, we compared the ability of 63 plant-beneficial phenazine-producing Pseudomonas strains representative of the worldwide diversity to inhibit the growth of three major potato pathogens: the oomycete Phytophthora infestans, the Gram-positive bacterium Streptomyces scabies, and the ascomycete Verticillium dahliae. The 63 Pseudomonas strains are distributed among four different subgroups within the P. fluorescens species complex and produce different phenazine compounds, namely, phenazine-1-carboxylic acid (PCA), phenazine-1-carboxamide (PCN), 2-hydroxyphenazine-1-carboxylic acid, and 2-hydroxphenazine. Overall, the 63 strains exhibited contrasted levels of pathogen inhibition. Strains from the P. chlororaphis subgroup inhibited the growth of *P*. *infestans* more effectively than strains from the P. fluorescens subgroup. Higher inhibition was not associated with differential levels of phenazine production nor with specific phenazine compounds. The presence of additional biocontrol-related traits found in P. chlororaphis was instead associated with higher *P*. *infestans* inhibition. Inhibition of *S. scabies* by the 63 strains was more variable, with no clear taxonomic segregation pattern. Inhibition values did not correlate with phenazine production nor with specific phenazine compounds. No additional synergistic biocontrol-related traits were found. Against V. dahliae, PCN producers from the P. chlororaphis subgroup and PCA producers from the P. fluorescens subgroup exhibited greater inhibition. Additional biocontrol-related traits potentially involved in V. dahliae inhibition were identified. This study represents a first step toward harnessing the vast genomic diversity of phenazine-producing Pseudomonas spp. to achieve better biological control of potato pathogens.

**IMPORTANCE** Plant-beneficial phenazine-producing Pseudomonas spp. are effective biocontrol agents, thanks to the broad-spectrum antibiotic activity of the phenazine antibiotics they produce. These bacteria have received considerable attention over the last 20 years, but most studies have focused only on the ability of a few genotypes to inhibit the growth of a limited number of plant pathogens. In this study, we investigated the ability of 63 phenazine-producing strains, isolated from a wide diversity of host plants on four continents, to inhibit the growth of three major potato pathogens: Phytophthora infestans, Streptomyces scabies, and Verticillium dahliae. We found that the 63 strains differentially inhibited the three potato pathogens. These differences are in part associated with the nature and the quantity of the phenazine compounds being produced but also with the presence of additional biocontrol-related traits. These results will facilitate the selection of versatile biocontrol agents against pathogens.

## INTRODUCTION

Potato (Solanum tuberosum L.) is an important food crop grown worldwide that contributes to feeding more than one billion people. While accounting for only 2% of the food energy supply (1), potato consumption has increased considerably in developing countries, and potato is regarded as a highly valuable crop that could significantly contribute to global food security (2). Approximately 21% of the attainable potato yield is lost to pathogens and viruses (3), and a large amount of money is spent to control these organisms, primarily by using synthetic pesticides (4, 5). For instance, using fungicides to control potato late blight, a disease caused by the oomycete Phytophthora infestans, has been estimated to represent an annual cost of 115.5 million euros in the Netherlands (5), which accounts for 14.7% of the total potato’s farm gate price (5). In addition, extensive use of most synthetic pesticides has deleterious consequences on human health and the environment (6, 7). Using naturally occurring bacteria that produce antimicrobial compounds capable of altering pathogen growth, such as plant-beneficial antibiotic-producing Pseudomonas spp., could represent a viable and environmentally friendly alternative and/or a complement to the use of synthetic pesticides (8, 9).

Plant-beneficial Pseudomonas spp. are ubiquitous rod-shaped Gram-negative bacteria that aggressively colonize the rhizosphere and protect the root system against soil-dwelling plant pathogens (8). Numerous strains produce antibiotic compounds, such as 2,4-diacetylphloroglucinol, pyrrolnitrin, and phenazines, which can inhibit pathogen growth in the rhizosphere (8, 10, 11). Phenazine compounds are particularly interesting because they display broad-spectrum antibiotic activity toward many fungal, oomycete, and bacterial plant pathogens (12, 13) and also because they promote survival and persistence of bacterial cells in the rhizosphere (11, 14). Phenazine production is mediated by a seven-gene operon (15), whose organization is conserved in all phenazine-producing Pseudomonas spp. sequenced to date (16, 17). The enzymes encoded by these biosynthetic genes catalyze the biosynthesis of phenazine-1-carboxylic acid (PCA), which is the first and main phenazine molecule produced by phenazine-producing pseudomonads. Some strains harbor additional biosynthetic genes, such as *phzH* and *phzO*, which enable them to produce additional phenazine molecules (18, 19). Strains harboring *phzH* produce phenazine-1-carboxamide (PCN) in addition to PCA (19), while strains harboring *phzO* produce, in addition to PCA, 2-hydroxphenazine (2-OH-PHZ) and 2-hydroxyphenazine-1-carboxylic acid (2-OH-PCA) (18). Interestingly, these four compounds do not seem to possess the same antimicrobial activity depending on the targeted plant pathogen. For example, the introduction of the *phzH* gene in two PCA-producing Pseudomonas strains enabled them to produce PCN and to suppress tomato foot and root rot caused by Fusarium oxysporum f. sp. *radicis*-*lycopersici* (19). Conversely, a *phzH* deletion mutant of P. chlororaphis subsp. *piscium* PCL1391 producing only PCA inhibited Verticillium dahliae microsclerotia germination more effectively than the wild-type strain producing PCN (19). This suggests that PCN has less activity toward V. dahliae microsclerotia than PCA. More recently, Yu et al. generated mutants of P. chlororaphis 30-84 producing different phenazine compounds (20). Their findings also support differences in antimicrobial activity for the different phenazine compounds.

Previously, our research group performed a comparative genomic analysis of 63 plant-beneficial phenazine-producing Pseudomonas strains isolated from a wide diversity of host plants on four continents (17). The 63 strains were distributed among four subgroups within the P. fluorescens species complex. The genomic diversity of these 63 strains was large, as reflected by the size of the pangenome, accounting for more than 25,000 protein-coding genes. We identified numerous phytobeneficial traits involved in plant pathogen suppression, plant growth promotion and rhizosphere colonization. The strains harbor a myriad of biocontrol-related traits, including type III and VI secretion systems and effectors, antibiotics, cyclic lipopeptides and siderophores. This diversity is interesting given that biocontrol-related traits could act in synergy to suppress plant diseases. For example, the importance of both phenazine and cyclic lipopeptide production by Pseudomonas sp. strain CMR12a in the biocontrol of Rhizoctonia solani has been clearly demonstrated (21). In other cases, the diversity of biocontrol-related traits could expand the biocontrol range of plant-beneficial Pseudomonas strains. For example, in strains producing both pyrrolnitrin and phenazines, phenazines have been shown to be more important for the biocontrol of Fusarium oxysporum (22) and *Sclerotium rolfsii* (23), while pyrrolnitrin has been shown to be more important for the biocontrol of Fusarium graminearum (24) and Sclerotinia sclerotiorum (25). The ability to produce these two compounds likely allows biocontrol strains to inhibit more plant pathogens than strains producing only one. Given that the biocontrol-related traits are unevenly distributed among the 63 strains under study, they will likely differ in their ability to inhibit plant pathogen growth.

The aim of this study was to characterize the abilities of 63 plant-beneficial phenazine-producing Pseudomonas strains to inhibit the growth of three of the most important potato pathogens: the oomycete *P. infestans*, the Gram-positive bacterium Streptomyces scabies, and the ascomycete V. dahliae. Several studies have already identified different phenazine-producing Pseudomonas strains as promising biocontrol agents against these three potato pathogens (26–29), but the exact biocontrol mechanisms involved, as well as the implication of additional key determinants other than phenazine production, are not fully understood. Therefore, in this study, in addition to characterizing the biocontrol potential of the 63 strains and quantifying the amount of phenazine compounds produced in different growth media, we also highlighted several biocontrol-related traits that could synergistically contribute to the inhibition of the three potato pathogens. In parallel, we also investigated the differential effects of the phenazine compounds produced by the strains toward the three potato pathogens under study. Taken together, this information will contribute to more rapidly and more efficiently selecting phenazine-producing Pseudomonas spp. for biocontrol.

## RESULTS

### *In vitro* antagonism of phenazine-producing *Pseudomonas* spp.

**FIG 1:**
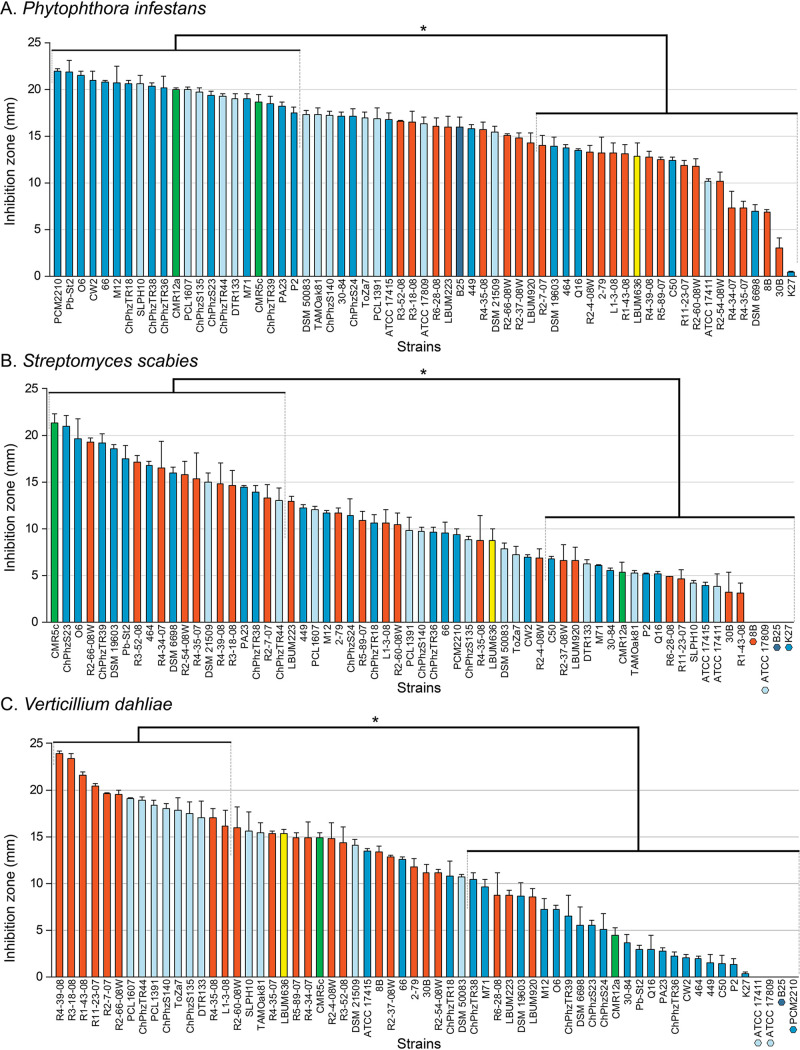
*In vitro* antagonism of plant-beneficial phenazine-producing Pseudomonas spp. (A) Inhibition of *Phytophthora infestans*. (B) Inhibition of *Streptomyces scabies*. (C) Inhibition of Verticillium dahliae. For the 63 strains under study, the inhibition zone, defined as the distance between the edges of the bacterial colonies and the pathogen vegetative tissues, was measured. The colors used for each strain correspond to the following phylogenetic groups: P. fluorescens subgroup (orange), *P*. *gessardii* subgroup (yellow), CMR12a/CMR5c subgroup (green), and P. chlororaphis subgroup (light blue for the *phzH*^+^ strain, blue for the *phzO*^+^ strain, and dark blue for B25, which does not harbor *phzH* nor *phzO*). When it was not possible to display the phylogenetic affiliation of the strain on the histogram bar, a colored symbol was added next to its name. Statistical analyses (Kruskal-Wallis test, followed by *post hoc* tests) discriminated two groups of strains significantly different from each other (*P* < 0.05). Error bars represent the standard errors.

The ability of 63 plant-beneficial phenazine-producing Pseudomonas strains to inhibit the growth of *P. infestans*, *S. scabies*, and V. dahliae was evaluated using confrontation assays. For each of the three potato pathogens, two groups of Pseudomonas strains were statistically discriminated: one group encompassing the strains showing the highest pathogen suppression activity and another group composed of strains showing the lowest pathogen suppression activity. The results are presented in Fig. 1. In general, the 63 phenazine-producing Pseudomonas strains efficiently inhibited the growth of the oomycete *P. infestans* on V8 agar (Fig. 1A), with the width of the inhibition zones ranging from 0.5 to 22 mm (median, 16.4 mm). However, on average, strains from the P. chlororaphis and the CMR12a/CMR5c subgroups inhibited *P*. *infestans* mycelial growth more effectively than the strains belonging to the two other subgroups. In fact, the strains showing the highest activity all belong to the P. chlororaphis and the CMR12a/CMR5c subgroups (Fig. 1A). Against the bacterial pathogen *S. scabies*, the 63 strains showed contrasting pathogen suppression activity on oat bran agar (OBA) (Fig. 1B), with inhibition zones ranging from 0 mm to 21.4 mm (median of 9.7 mm) and no clear taxonomic segregation. Against V. dahliae, the 63 strains also showed contrasting pathogen suppression activity on potato dextrose agar (PDA) (Fig. 1C), with inhibition zones varying between 0 and 23.9 mm (median, 11.2 mm). The width of these inhibition zones, however, showed a clear segregation between P. chlororaphis strains harboring the phenazine biosynthetic gene *phzH* (responsible for the production of PCN) and those harboring *phzO* (responsible for the production of 2-OH-PHZ and 2-OH-PCA). While most *phzH^+^* strains were among the group showing the highest antagonistic activity, strains harboring *phzO* consistently showed very low activity against V. dahliae. Several PCA-producing strains from the P. fluorescens subgroup also exhibited very high pathogen suppression activity toward V. dahliae.

### Phenazine quantification in King’s B broth.

**FIG 2:**
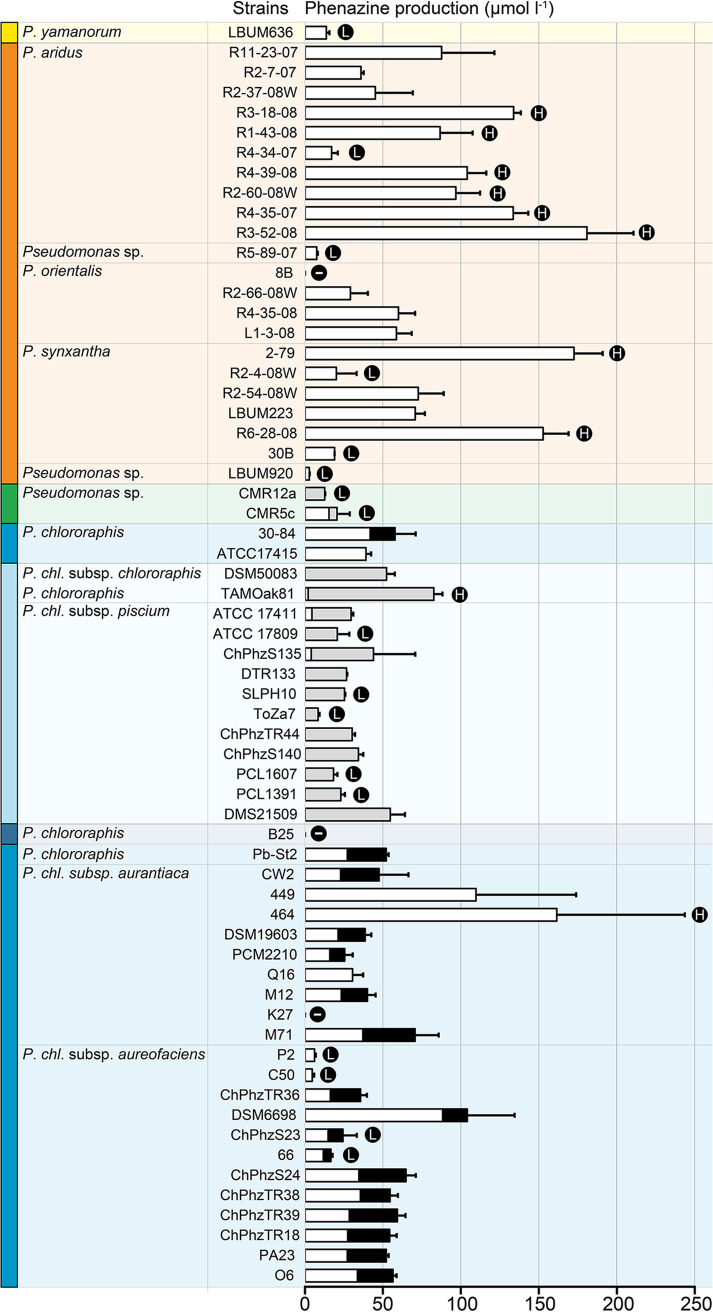
Phenazine production in King’s B broth by the 63 strains under study. Three phenazine compounds (PCA, PCN, and 2-OH-PHZ) were quantified from 5-day-old KB broth cultures using HPLC. The colors correspond to PCA (white), PCN (gray), and 2-OH-PHZ (black). The symbol “–” indicates the absence of phenazine detection. Phenazine production by the different strains was compared between each other using Kruskal-Wallis and *post hoc* tests. Two groups of strains were statistically discriminated, one group encompassing the strains with high phenazine production (H) and the other encompassing the strains with low phenazine production (L). Error bars represent the standard errors. The colors used for each strain correspond to the following phylogenetic groups: P. fluorescens subgroup (orange), *P*. *gessardii* subgroup (yellow), CMR12a/CMR5c subgroup (green), and P. chlororaphis subgroup (blue).

As a first step in understanding the differences observed in pathogen inhibition between the 63 strains, three different phenazine compounds (PCA, PCN, and 2-OH-PHZ) were quantified in King’s B (KB) broth using high-performance liquid chromatography (HPLC) following a 5-day standardized growth period. 2-OH-PCA was not quantified because of the unavailability of a standard for reliable HPLC quantification. The results are presented in Fig. 2. At least one phenazine compound (PCA, PCN, or 2-OH-PHZ) was detected in the growth medium of 60 strains out of 63, at concentrations ranging from 2.8 to 181 μmol liter^−1^. No phenazine compound of any kind was detected in the growth medium of strains 8B, B25, and K27. Two groups of strains were statistically discriminated: one group encompassing the strains producing the largest amount of phenazine compounds and another group composed of strains producing the smallest amount of phenazine compounds. In strains belonging to the P. fluorescens subgroup (22 strains) and to the *P*. *gessardii* subgroup (1 strain), only PCA was detected at concentrations ranging from 2.8 to 181 μmol liter^−1^. Eight out of ten strains producing the largest amount of phenazines belong to the P. fluorescens subgroup. PCN was detected in the 15 P. chlororaphis strains harboring *phzH*, at concentrations ranging from 5.1 to 80.5 μmol liter^−1^. In 11 out of 15 strains producing PCN, no PCA was detected. For the four other strains, PCA represented less than 20% of the total amount of phenazine compounds being produced, except for CMR5c, which produced four times more PCA than PCN. Conversely, for strains harboring *phzO* (except K27), PCA was always detected in the growth medium and in greater amounts than 2-OH-PHZ. 2-OH-PHZ was not detected in seven *phzO*^+^ strains, while it was detected in the other *phzO*^+^ strains at concentrations ranging from 4.9 to 33.5 μmol liter^−1^. The 26 *phzO* nucleotide sequences were compared. However, we did not find any mutation that could explain why these seven strains do not produce 2-OH-PHZ under the growth conditions used in this study.

### Correlation between phenazine production and pathogen inhibition.

**FIG 3:**
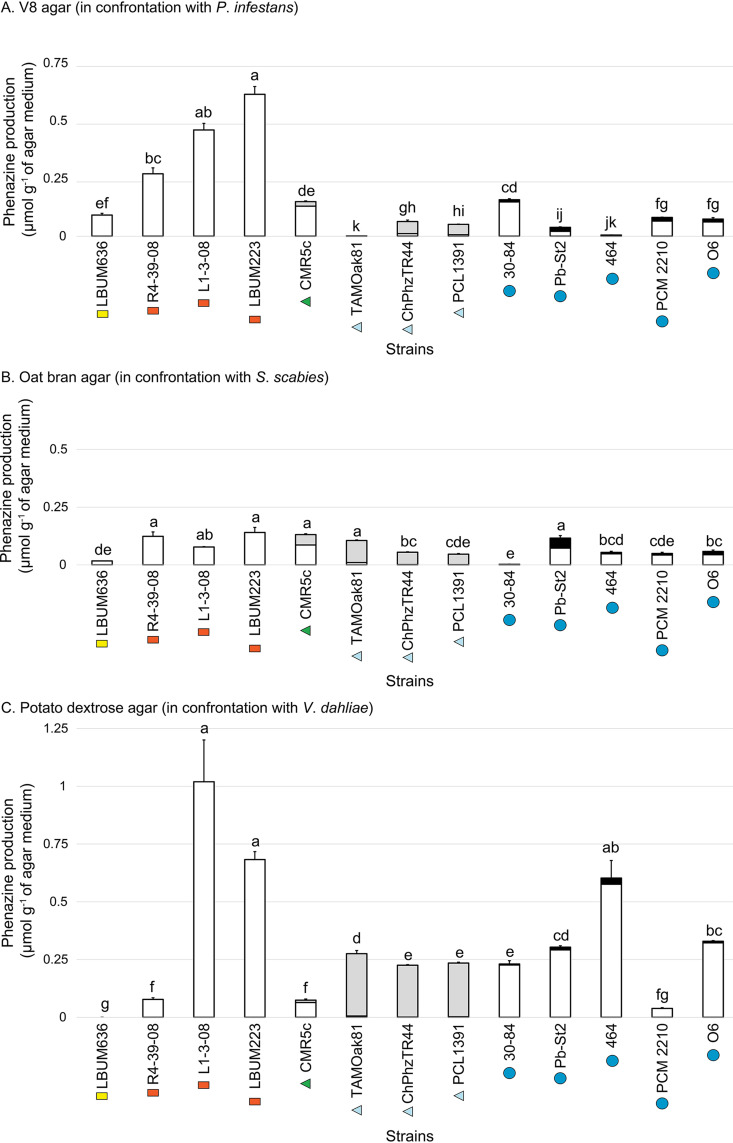
Phenazine production in three different agar-solidified growth media. Three phenazine compounds (PCA, PCN, and 2-OH-PHZ) were quantified in V8 agar, OBA, and PDA in the presence of the three potato pathogens. The histogram bars correspond to the amount of PCA (white), PCN (gray), and 2-OH-PHZ (black) being produced. For each medium, strains with different letters are significantly different (*P* < 0.05). Phenazine production was not compared across the different media. Error bars represent the standard errors. For each strain, a symbol indicates the phenazine compounds likely to be produced based on the presence or absence of the two accessory phenazine biosynthetic genes in their genome: PCA producer (rectangle), PCN (and PCA) producer (triangle), and 2-OH-PHZ (and PCA) producer (circle). The color of each symbol specifies the phylogenetic group to which the strain belongs: P. fluorescens subgroup (orange), *P*. *gessardii* subgroup (yellow), CMR12a/CMR5c subgroup (green), and P. chlororaphis subgroup (blue).

While the phenazine quantification data obtained in the previous section provided much-needed insights into the phenazine production potential of the 63 strains under study, the quantification of phenazine production under the same conditions used for the confrontation assays was necessary to better understand the differential inhibition exhibited by the strains. Thirteen representative strains were selected and grown in the presence of the potato pathogens on the three media used for the confrontation assays (V8 agar, OBA, and PDA). The amount of phenazine compounds in the medium was quantified by liquid chromatography-coupled mass spectrometry (LC-MS). The results are presented in Fig. 3. On V8 agar in the presence of *P*. *infestans*, phenazine compounds were detected in the medium for every strain, at concentrations ranging from 0.001 to 0.61 μmol g^−1^ of medium. In this medium, the three strains from the P. fluorescens subgroup stand out for their high phenazine production. On OBA in the presence of *S*. *scabies*, phenazine compounds were detected for every strain, at concentrations ranging from 0.002 to 0.14 μmol g^−1^ of medium. These values are mostly lower than the amounts of phenazine compounds detected on V8 agar, except for some strains belonging to the P. chlororaphis subgroup. On PDA and in the presence of V. dahliae, phenazine compounds were detected for every strain except LBUM636, at concentrations ranging from 0.04 to 1.02 μmol g^−1^ of medium.

**FIG 4:**
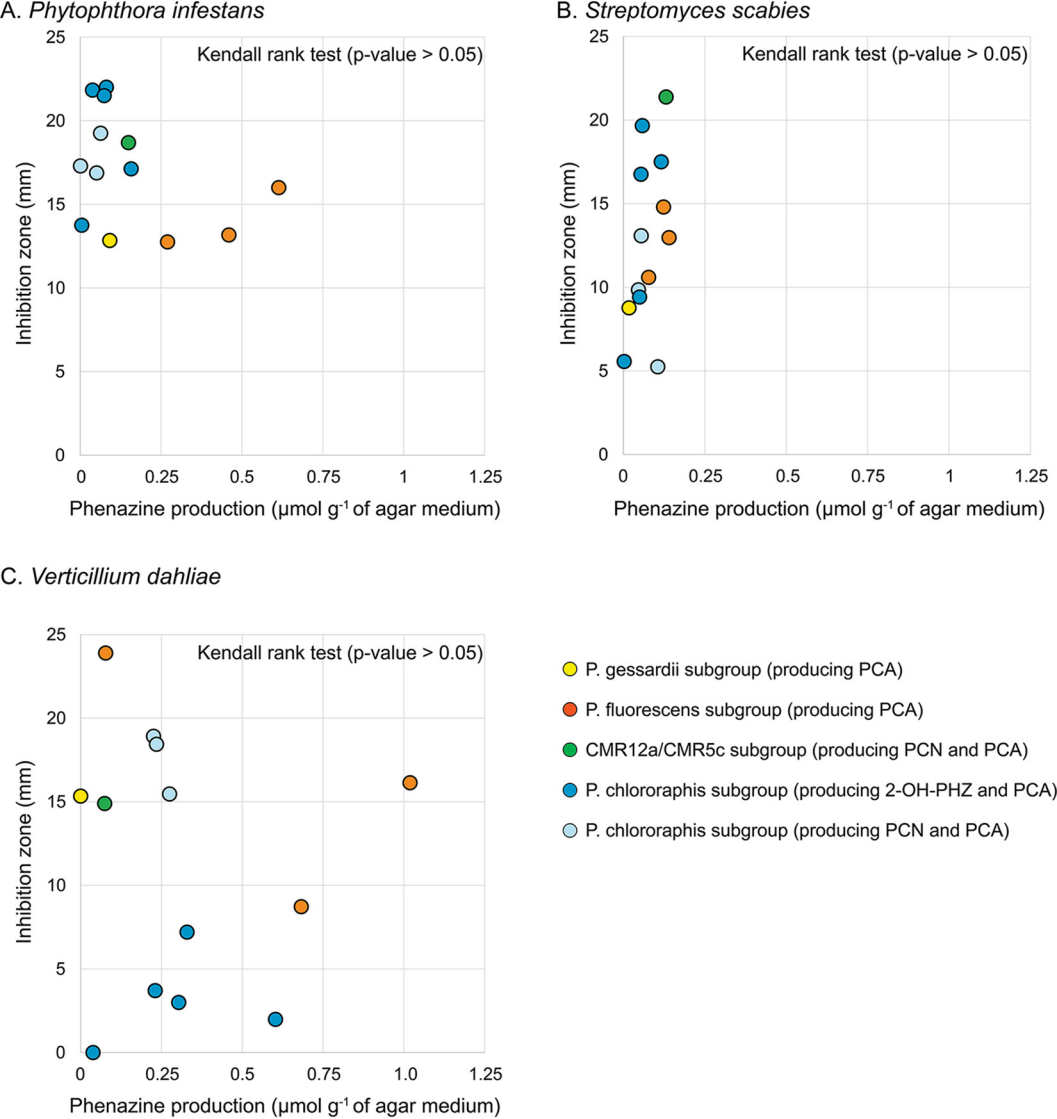
Correlation between pathogen inhibition and total phenazine production by Pseudomonas spp. (A) Phytophthora infestans. (B) Streptomyces scabies. (C) Verticillium dahliae. For each pathogen, correlations between the width of the inhibition zones and phenazine production in the confrontation medium was examined using Kendall rank tests.

To visualize whether the higher levels of phenazine production exhibited by some strains were associated with higher pathogen inhibitions, the width of the inhibition zones obtained previously were plotted against the total amount of phenazine compounds being produced by the 13 Pseudomonas strains (Fig. 4). The widths of the inhibition zones were also plotted separately against the amounts of PCA/PCN/2-OH-PHZ being produced (see Fig. S1 in the supplemental material). The Kendall rank correlation coefficient *τ* was calculated for the three pathogens to evaluate if the total amount of phenazine compounds being produced correlates with the size of the inhibition zones. The correlation coefficient was close to zero for P. infestans, S. scabies, and V. dahliae inhibition data, and each test was nonsignificant (*P* > 0.05). This indicates that, overall, the amount of phenazine compounds being produced by the 13 Pseudomonas strains does not correlate with their ability to inhibit the three potato pathogens.

10.1128/mSphere.00427-21.1 FIG S1 Correlation between pathogen inhibition by Pseudomonas spp. and the amount of the three phenazine molecules produced in the three media used for the confrontation assays. For each pathogen, correlation between the width of the inhibition zones and phenazine production was studied using Kendall rank tests (*P* < 0.05). For each phenazine molecule, strains that do not produce it were removed from the analyses. Download FIG S1, DOCX file, 0.7 MB. © Crown copyright 2021. 2021 Crown https://creativecommons.org/licenses/by/4.0/ This content is distributed under the terms of the Creative Commons Attribution 4.0 International license.

**FIG 5:**
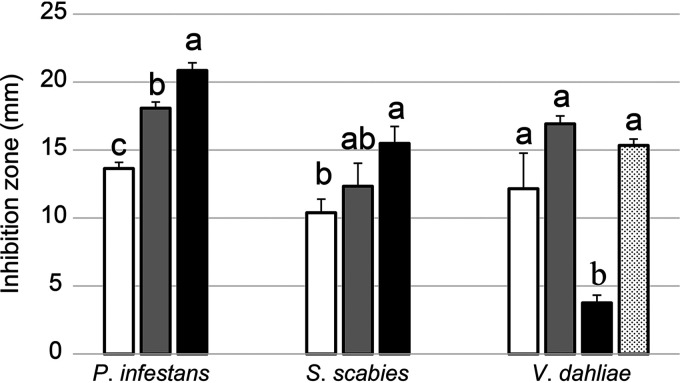
Mean inhibition of the three studied potato pathogens by phenazine-producing Pseudomonas spp. The 13 strains were grouped according to the phenazine compounds they produce in the three media used for the confrontation assays. The colors correspond to strains producing only PCA (white), strains producing PCN (gray), strains producing 2-OH-PHZ (black), and strains producing no phenazine compound of any kind (dotted). For each pathogen, groups with different lowercase letters are significantly different (*P *<* *0.05). Groups were not compared across pathogens. Error bars represent the standard errors.

The 13 strains were grouped according to the type of phenazine compounds they produce (Fig. 5). On average, strains producing PCN or 2-OH-PHZ inhibited *P*. *infestans’* growth significantly better than strains solely producing PCA (Fig. 5A). On the other hand, strains producing PCN and strains producing solely PCA tended to inhibit V. dahliae better than strains producing 2-OH-PHZ (Fig. 5C). There was no difference for *S. scabies*, except between strains producing PCA and strains producing 2-OH-PHZ (Fig. 5B).

### Antibiotic activity of phenazine compounds.

**FIG 6:**
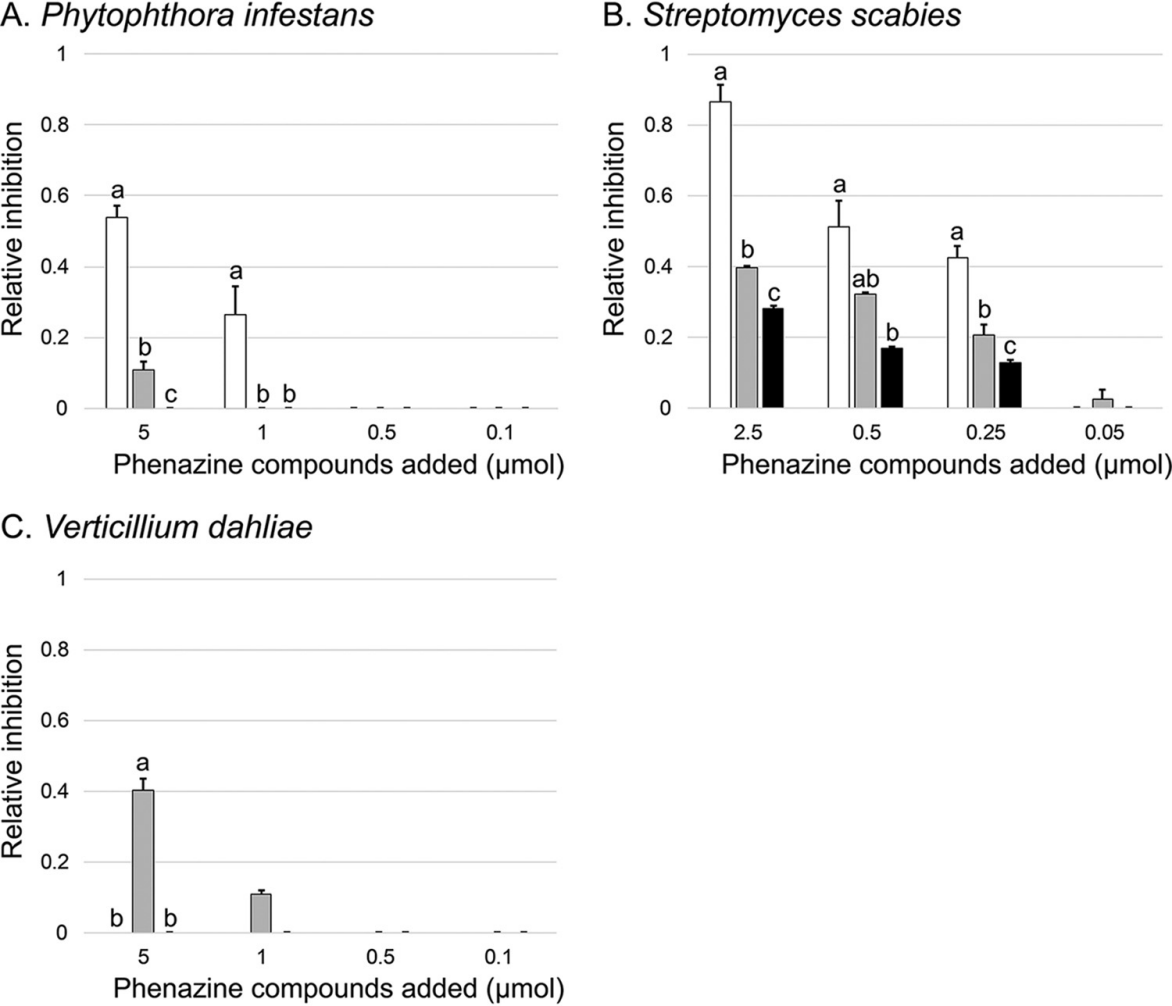
Antibiotic activities of PCA, PCN, and 2-OH-PHZ against three potato pathogens. Phenazine compounds dissolved in DMSO were added to culture media inoculated with one of the three potato pathogens: *Phytophthora infestans* (A), *Streptomyces scabies* (B), and Verticillium dahliae (C). The colors correspond to PCA (white), PCN (gray), and 2-OH-PHZ (black). Statistical analyses (Kruskal-Wallis, followed by *post hoc* tests) were conducted to compare each concentration, and significant differences (*P* < 0.05) are indicated with different lowercase letters. Relative inhibitions were not compared across different concentrations or pathogens. Error bars represent the standard errors.

To further evaluate whether the differences in terms of pathogen inhibition were related to the type of phenazine compounds being produced, inhibition tests with purified phenazine compounds were carried out. The goal was to establish whether the different phenazine molecules (PCA, PCN, and 2-OH-PHZ) have differential antibiotic activity against the three studied potato pathogens. The results are presented in Fig. 6. Against *P. infestans*, PCA was shown as the most effective phenazine compound to inhibit mycelial growth. The addition of 5 μmol of PCA (two spots of 2.5 μmol of PCA deposited at each edge of the petri dishes) led to a high inhibition of mycelial growth, while 0.5 μmol did not have any effect (Fig. 6A). Of the three pathogens under study, *S. scabies* was the most susceptible to phenazine antibiotics. We found that 2.5 μmol of PCA deposited in the center of a petri dish nearly inhibited all growth of *S. scabies* inoculated over the whole surface (Fig. 6B). In contrast, PCN and 2-OH-PHZ also exhibited antibiotic activity but to a lower level than PCA. For V. dahliae, only PCN was mildly inhibitory (Fig. 6C).

### Additional biocontrol-related traits potentially involved in pathogen suppression.

**FIG 7:**
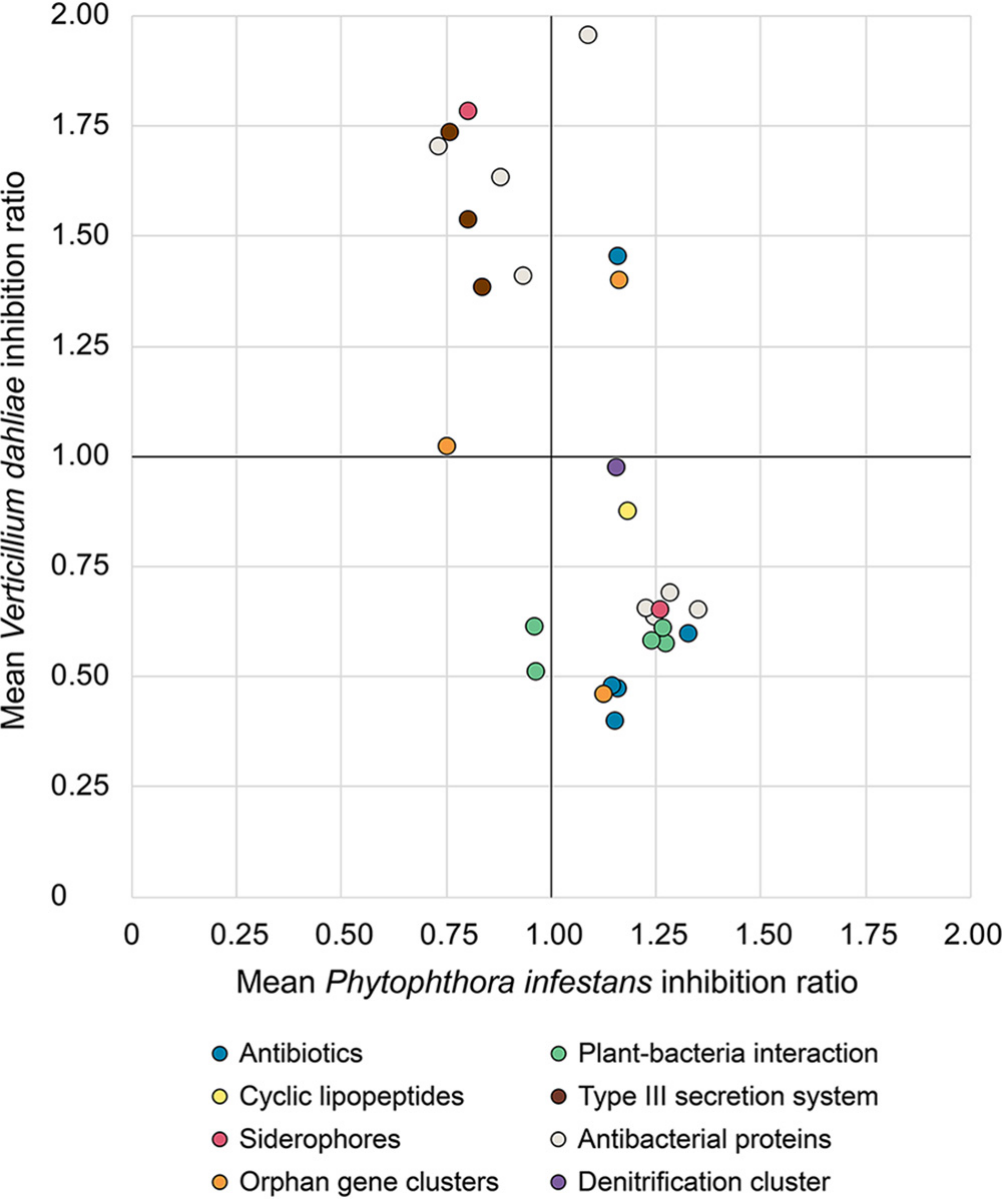
Mean pathogen inhibition ratios associated with the presence or absence of phytobeneficial traits. Ratios for each phytobeneficial trait were calculated by dividing the mean inhibition achieved by Pseudomonas strains harboring it by the mean inhibition achieved by strains not harboring it. For example, if a gene/cluster has a ratio of 1.5 for a given pathogen, it means that strains harboring this gene/cluster inhibit the pathogen 50% more than strains not harboring it. Only phytobeneficial traits that significantly correlate (*P* < 0.05) with higher or lower pathogen inhibition and are present in five strains or more are presented.

In addition to phenazine antibiotics, we wanted to determine whether other determinants could potentially be involved in pathogen growth suppression. Previously, we studied the distribution of genes/clusters involved in biocontrol, plant-growth promotion and rhizocompetence in the 63 Pseudomonas strains under study (17). These results were used together with the inhibition data to search for genes and clusters whose presence correlates with high or low pathogen inhibition. For each of the 74 genes/clusters of interest and for each pathogen, we calculated the mean inhibition for the strains harboring the gene/cluster and compared it to the mean inhibition for strains that do not harbor it (using Wilcoxon-Mann-Whitney test). We then calculated a mean inhibition ratio by dividing these two means. If this ratio is superior to 1, it means that strains harboring the gene/cluster tend to be more antagonistic than strains that do not have it. Ratios associated with their respective *P* values are presented in Fig. 7 and Table 1. We found 21 genes/clusters associated with higher or lower *P*. *infestans* inhibition, with mean inhibition ratios ranging from 0.74 to 1.35. We did not find any determinant that could be significantly associated with differential inhibition for *S*. *scabies*. For V. dahliae, 24 genes/clusters were associated with differential inhibition, with mean inhibition ratios ranging from 0.40 to 1.96. No common determinant was significantly associated with higher pathogen inhibition for both P. infestans and V. dahliae.

**TABLE 1:** Phytobeneficial traits associated with *P*. *infestans* or V. dahliae differential inhibition by phenazine-producing Pseudomonas strains^*a*^

Gene/cluster	Category	Phytophthora infestans		Verticillium dahliae	
		*P*	MI ratio	*P*	MI ratio
Triglyceride lipase	Antibacterial protein	>0.05	1.09	2.19E–03	1.96
Unknown siderophore 1	Siderophore	2.61E–03	0.80	3.96E–04	1.78
Hrp1	Type III secretion system	2.88E–05	0.76	7.35E–04	1.74
Putative Rhs toxin	Antibacterial protein	1.38E–04	0.74	3.16E–03	1.70
*phzO*	Antibiotic	6.17E–03	1.15	1.49E–06	0.40
Pore-forming pyocin	Antibacterial protein	>0.05	0.88	2.10E–02	1.63
Type III secretion system	Type III secretion system	1.56E–04	0.80	1.01E–02	1.54
HCN	Antibiotic	1.79E–05	1.33	1.90E–03	0.60
PAA catabolism	Plant-bacterium interaction	7.78E–05	1.28	8.26E–04	0.57
Tae4-like	Antibacterial protein	3.37E–05	1.35	1.04E–02	0.65
Pyrrolnitrin	Antibiotic	6.56E–03	1.16	1.12E–05	0.47
HPR	Antibiotic	9.60E–03	1.15	3.37E–05	0.48
NRPS 9	Orphan gene cluster	>0.05	1.13	4.42E–04	0.46
2,3-btd biosynthesis	Plant-bacterium interaction	1.82E–03	1.24	1.17E–03	0.58
iaa biosynthesis	Plant-bacterium interaction	1.86E–04	1.27	3.10E–03	0.61
*phzH*	Antibiotic	>0.05	1.16	1.45E–02	1.45
DUF4150/Tox-GHH2	Antibacterial protein	2.89E–04	1.25	8.14E–03	0.64
Achromobactin	Siderophore	1.73E–04	1.26	1.15E–02	0.65
Tox-REase-5	Antibacterial protein	5.03E–04	1.29	2.64E–02	0.69
S-type pyocin (DNase)	Antibacterial protein	5.34E–04	1.23	1.37E–02	0.66
NRPS-PKS 5	Orphan gene cluster	>0.05	1.16	3.94E–02	1.40
SPI-1 (Inv/Mixi/Spa)	Type III secretion system	2.72E–02	0.84	>0.05	1.38
iaa catabolism	Plant-bacterium interaction	>0.05	0.97	6.74E–03	0.51
Rhs protein (nuclease)	Antibacterial protein	>0.05	0.93	3.41E–02	1.41
Acetoin catabolism	Plant-bacterium interaction	>0.05	0.96	5.99E–03	0.61
Viscosin	Cyclic lipopeptides	3.10E–02	1.19	>0.05	0.87
NRPS 2	Orphan gene cluster	4.28E–02	0.75	>0.05	1.02
Nor	Denitrification cluster	1.33E–02	1.16	>0.05	0.97

a Only phytobeneficial traits that significantly correlate (*P* < 0.05) with higher or lower pathogen inhibition in at least one of the two pathogens under study and are present in five Pseudomonas strains and more are presented. MI ratio, mean inhibition ratio.

## DISCUSSION

In this study, we compared the ability of 63 phenazine-producing Pseudomonas strains isolated from a wide diversity of host plants on four continents (17) to inhibit the growth of three different potato pathogens of economic significance: the oomycete *P. infestans*, the Gram-positive bacterium *S. scabies*, and the ascomycete V. dahliae. We also measured the amount of phenazine molecules being produced by 13 representative strains in the confrontation assay media, to determine whether the differences observed in pathogen inhibition correlate with different phenazine production levels or whether the capacity to produce a specific phenazine compound correlates with high or low inhibition values. In parallel, the antibiotic activity of different concentrations of the purified phenazine molecules under study was also measured. Finally, pathogen inhibition values were analyzed in light of genomic data obtained in a previous study (17) to identify positive or negative associations between pathogen inhibition and the presence of specific phytobeneficial traits.

Phenazine antibiotics have been previously shown to inhibit the vegetative growth of *P*. *infestans* (27, 30) and other oomycetes (31, 32). Notably, we established in a previous study that an isogenic mutant of *P*. *yamanorum* LBUM636 impaired in phenazine production inhibited the growth of *P*. *infestans* less effectively than the wild type (27). It is therefore not surprising that nearly every strain under study inhibited the growth of *P*. *infestans*, to a certain extent. However, on average, strains from the P. chlororaphis and CMR12a/CMR5c subgroups performed better than strains from the P. fluorescens subgroup (Fig. 1). We did not find any correlation between the level of phenazine production and the width of the inhibition zones observed for *P*. *infestans* (Fig. 4), suggesting that the differences in *P*. *infestans* inhibition do not originate from differences in phenazine production between the different strains. In fact, on V8 agar, the three strains from the P. fluorescens subgroup produced much more phenazine compounds than any other strains (Fig. 3). On the other hand, while strains from the P. fluorescens subgroup only produce PCA, most P. chlororaphis strains produce additional phenazine compounds, namely, 2-OH-PHZ and PCN. PCN producers and 2-OH-PHZ producers inhibited *P*. *infestans* more, on average, than strains producing only PCA (Fig. 5). This suggests that the differences in *P*. *infestans* growth inhibition could originate from the type of phenazine compounds being produced. Nonetheless, when the three purified phenazine compounds were tested for their antibiotic activity against *P*. *infestans*, PCA had the highest activity, whereas PCN had very low activity, and 2-OH-PHZ no detectable activity at all (Fig. 6). In addition, the amounts of purified phenazine compounds added to the agar-solidified media were comparable to the amounts produced and quantified in these media when phenazine-producing Pseudomonas spp. were present. Together, these findings suggest that, whereas PCA probably plays an important role in the inhibition of *P*. *infestans* for PCA-producing strains, strains from the P. chlororaphis and CMR12a/CMR5c subgroups likely rely on other biocontrol-related traits to inhibit the growth of *P*. *infestans*. Fourteen phytobeneficial traits were found to be associated with greater *P*. *infestans* inhibition when present in the strains under study (Table 1). This includes biosynthetic clusters involved in antibiotic production (hydrogen cyanide, pyrrolnitrin, and 2-hexyl 5-propyl resorcinol), cyclic lipopeptide production (viscosin), and siderophore production (achromobactin), as well as several antibacterial proteins. Most of these genes/clusters are present in almost all strains belonging to the P. chlororaphis subgroup but not outside this subgroup. While their association with a greater *P*. *infestans* inhibition may only originate from their taxonomic distribution, some are likely to contribute to *P*. *infestans* growth suppression. For example, every strain belonging to the P. chlororaphis and CMR12a/CMR5c subgroups harbors the *hcnABC* gene cluster (17), which is responsible for the production of the respiratory toxin hydrogen cyanide (HCN) (33). In our study, this cluster was associated with a higher pathogen inhibition by the strains harboring it (+33%), and several P. chlororaphis strains under study are known to produce HCN (34–36). *P*. *infestans* has been previously reported to be susceptible to this molecule (37). However, in a recent study, HCN was shown to play only a minor role in the inhibition of *P*. *infestans* mycelial growth by the phenazine-producing strain R47 (38). This strain belongs to P. chlororaphis subsp. *aureofaciens* and is closely related to several strains under study. Reverse genetic approaches will need to be used to evaluate the importance of these biocontrol-related traits in the inhibition of *P*. *infestans* growth.

The phenazine-producing strain *P. synxantha* LBUM223 has previously been shown to inhibit *S. scabies* growth *in vitro* (28) and to reduce potato common scab symptoms under soil conditions, both in growth chambers (28) and in the field (29). In our assay, LBUM223-mediated *S*. *scabies* growth inhibition was intermediate, with a mean inhibition zone of 13 mm. Twenty strains exhibited higher inhibition against *S*. *scabies* than LBUM223, with inhibition zones ranging from 13.1 to 21.4 mm (Fig. 1). Unlike *P*. *infestans* inhibition, there was no clear phylogenetic pattern, with strains from three different subgroups included in the high inhibition group (Fig. 1). In addition, we did not find any correlation between the total amount of phenazine compounds being produced and the width of the inhibition zones (Fig. 4). PCA has already been identified as an important determinant in *S*. *scabies* growth inhibition by *P*. *synxantha* LBUM223 (28). Indeed, an isogenic mutant of *P*. *synxantha* LBUM223 impaired in phenazine production was shown to inhibit *S*. *scabies* growth and to protect potato plants against common scab less effectively than the wild type (28, 39). In this study, the addition of 2.5 μmol of PCA was shown to inhibit the growth of *S*. *scabies* drastically (Fig. 6), and we found that the two other phenazine compounds, PCN and 2-OH-PHZ, inhibited *S*. *scabies* growth, even though PCA inhibitory activity was higher (Fig. 6). Based on these results, the amount of phenazine compounds being produced by several strains in OBA (Fig. 3) are sufficient to inhibit *S*. *scabies* growth. Interestingly, the three strains that did not produce any phenazine compounds in KB broth under our conditions (although they harbor the *phz* biosynthetic operon) had no effect on *S*. *scabies* growth, reinforcing the importance of phenazine production for *S*. *scabies* growth inhibition. Unlike *P. infestans* inhibition, we did not find any phytobeneficial trait that correlates with a high *S*. *scabies* growth inhibition, which is partly related to the absence of phylogenetic patterns in the differential inhibition exhibited by the 63 strains. However, this does not mean that other biocontrol-related traits not highlighted in this study but found in some strains do not play a role in *S*. *scabies* inhibition.

The 63 strains exhibited major differences in their ability to inhibit V. dahliae growth. The group displaying the highest inhibition was composed of PCN-producing strains from the P. chlororaphis subgroup and PCA-producing strains from the P. fluorescens subgroup (Fig. 1). Conversely, 2-OH-PHZ-producing strains were particularly inefficient at inhibiting V. dahliae growth, even though many of those strains were good at inhibiting the growth of *P*. *infestans* and *S*. *scabies*. We did not find any correlation between total phenazine production and inhibition values (Fig. 4). However, we found that the three phenazine molecules display different inhibitory activity. Only PCN inhibited mycelial growth under our conditions (Fig. 6). These results differ from those obtained by Debode et al. (26). These authors found that a PCL1391 mutant impaired in PCN production and producing only PCA inhibited V. dahliae microsclerotia germination slightly more effectively than did the wild type. Furthermore, a PCL1391 mutant impaired in phenazine production was as effective as the wild type in inhibiting microsclerotia germination. We hypothesize that this discrepancy regarding PCN antibiotic activity might originate from the physiological differences between actively growing mycelium and the melanized resting structures that are microsclerotia. While the antibiotic activity of PCN might explain, to a certain extent, why *phzH*^+^ strains are efficient at inhibiting mycelial growth, it does not explain the higher inhibition exhibited by PCA-producing strains, considering that PCA had no antibiotic activity under our conditions. This suggests that other biocontrol-related traits are likely involved in the high V. dahliae inhibition by the strains belonging to the P. fluorescens subgroup. Nine phytobeneficial traits were found to be associated with a higher V. dahliae inhibition (Table 1). For example, strains harboring at least one copy of a gene, identified previously (17) and encoding a putative type VI effector with a triglyceride lipase domain (PF01764), were 96% more effective in inhibiting mycelial growth than strains not harboring it (Table 1). However, given that this effector is likely injected in neighboring cells using the type VI secretion system, it is unlikely to play a role under our conditions. Similarly, type III secretion systems and antibacterial proteins are unlikely to be involved in pathogen growth inhibition under our conditions, even though these phytobeneficial traits are associated with a higher inhibition. These associations rather originate from their taxonomic distribution. Strains from the P. fluorescens subgroup harbor a cluster involved in the biosynthesis of an undescribed siderophore (17). This siderophore is associated with a higher inhibition (+78%). This siderophore could be involved in V. dahliae inhibition as siderophores have already been demonstrated to engage in V. dahliae inhibition (40). Finally, the orphan gene cluster NRPS-PKS 5 was also associated with a higher inhibition (+40%) for strains harboring it. This cluster is only found in strains from the *piscium* subspecies and might contribute, in addition to PCN, to the superior inhibition exhibited by PCN-producing strains from the P. chlororaphis subgroup.

It is possible that phenazine production by *phzO*^+^ Pseudomonas strains was slightly underestimated in our study given that 2-OH-PCA was not quantified because of the unavailability of a standard for reliable HPLC quantification. However, we believe that the total amount of 2-OH-PCA is likely negligible compared to the amount of PCA and 2-OH-PHZ produced by the strains. The conversion of 2-OH-PCA from PCA is mediated by the flavin-diffusible monooxygenase PhzO (18). 2-OH-PCA subsequently undergoes a spontaneous decarboxylation leading to the production of 2-OH-PHZ (18, 41). Several authors have reported a nearly total conversion of 2-OH-PCA to 2-OH-PHZ in solutions buffered at pH 7 (18, 41), which is close to the pH of the different media used in our study. Nevertheless, several media used in this study (PDA and OBA) are not buffered, and it is possible that the growth of the pathogen/biocontrol agent resulted in the acidification of the growth medium. In this case, the conversion of 2-OH-PCA to 2-OH-PHZ would remain incomplete.

In this study, phenazine production was quantified in four different media, namely, KB broth, V8 agar, OBA, and PDA. A considerable variability in the amounts of phenazine compounds being produced by the 13 strains across the different media used for the confrontation assays was observed. This was expected, considering the differences in the growth media composition. Indeed, phenazine production has been shown to fluctuate greatly depending on many environmental factors, such as pH, temperature and the presence of glucose or amino acids (42–44). For example, phenazine production is increased in *P*. *synxantha* 2-79, in P. chlororaphis subsp. *aureofaciens* O6 and in P. chlororaphis subsp. *piscium* PCL1391 in the presence of glucose (42, 43, 45). In addition, it has been shown that phenazine-producing Pseudomonas strains can be affected differently by the presence of some compounds (42). This explains why phenazine production for some strains appears to remain stable across the different media, while for others it varies substantially. In addition, some factors, such as growth time, may impact the amount of phenazine compounds being quantified. For example, the phenazine-producing Pseudomonas strains were incubated in the confrontation media for different periods of time, ranging from 6 days on OBA to 20 days on PDA. It is possible that the incubation time influenced phenazine production, resulting in the accumulation of more or less phenazine compounds in the growth media. In addition, it is also possible that the presence of the potato pathogens in the petri dish could influence phenazine production. For example, several soilborne *Streptomyces* strains have been reported to interfere with quorum-sensing signaling in the phenazine-producing opportunistic pathogen Pseudomonas aeruginosa, leading to a reduction in phenazine production (46, 47). In addition, fusaric acid produced by the ascomycete Fusarium spp. has been shown to inhibit PCN production by P. chlororaphis subsp. *piscium* PCL1391, even under conditions that are favorable to its production (42, 48). It might be possible that similar mechanisms are used by the three potato pathogens under study to interfere with phenazine production. These all represent interesting research questions to address in future studies.

In this study, we identified several plant-beneficial phenazine-producing Pseudomonas strains that excel in inhibiting the growth of some of the three potato pathogens under study. However, P. chlororaphis subsp. *piscium* ChPhzTR44, a PCN-producing strain isolated from the rhizosphere of tomato grown in the Fusarium wilt suppressive soils of Châteaurenard in France (49), is the only strain included in the higher inhibition group for all three potato pathogens under study (Fig. 1). In addition to the phenazine biosynthetic operon, this strain harbors the HCN biosynthetic cluster *hcnABC*, the viscosin biosynthetic cluster, three siderophore biosynthetic clusters and two orphan NRPS/NRPS-PKS gene clusters (17). Moreover, this strain was shown to be a good colonizer of the potato rhizosphere, being detected by quantitative PCR at 6.54 × 10^6^ *phzD* copies per g of rhizosphere soil (50). This is interesting because biocontrol agent population size in the rhizosphere has been shown to correlate with disease incidence reduction (51, 52) and with antibiotic accumulation (53, 54). While this strain appears to be an excellent candidate to suppress the three different potato pathogens under study, further investigations in the presence of the plant, grown under controlled and field conditions, are needed to confirm its effectiveness as a versatile biocontrol agent.

In conclusion, we compared the ability of 63 phenazine-producing Pseudomonas strains to inhibit the growth of three potato pathogens of economic significance. Overall, the strains exhibited contrasted levels of pathogen inhibition and phenazine production. While several differences may be explained by the nature or the quantity of phenazine compounds being produced, our results suggest that other biocontrol traits are likely involved. Reverse genetic approaches will need to be applied in order to validate the implication of those biocontrol-related traits in pathogen suppression. This study represents a first step in harnessing the tremendous genomic diversity of phenazine-producing Pseudomonas spp. with the objective of strengthening biological control of potato pathogens.

## MATERIALS AND METHODS

### Bacterial strains and plant pathogens.

The 63 Pseudomonas strains used in this study are listed in Table 2. The strains were routinely grown in KB broth (55) at 25°C for 24 h under continuous shaking (120 rpm). Populations were estimated using spectrophotometer readings at 600 nm and standard curves. Three different pathogens were used*. Phytophthora infestans* (genotype US-8) was grown on 10% unclarified V8 (Campbell, Camden, NJ) agar plates at 20°C for 10 days. *Streptomyces scabies* (LBUM848) was grown on oat bran broth (56) at 28°C for 6 days with continuous shaking (200 rpm). Verticillium dahliae (717.96) was grown on PDA (BD, Franklin Lakes, NJ) at 25°C for 3 weeks.

**TABLE 2:** Pseudomonas strains used in this study

*Pseudomonas* strain(s)	Origin	Genome sequence (reference)	Reference(s) or source
P. yamanorum (*P*. *gessardii* subgroup)			
LBUM636	Strawberry rhizosphere, Canada	60	60
			
P. aridus (P. fluorescens subgroup)			
R11-23-07, R2-7-07, R2-37-08W, R3-18-08, R1-43-08, R4-34-07, R4-39-08, R2-60-08W, R4-35-07, R3-52-08	Wheat rhizosphere, USA	17	16, 61
			
P. orientalis (P. fluorescens subgroup)			
8B	Wheat rhizosphere, Iran	17	62
R2-66-08W, R4-35-08, L1-3-08	Wheat rhizosphere, USA	17	61
			
P. synxantha (P. fluorescens subgroup)			
2-79	Wheat rhizosphere, USA	17	63
LBUM223	Strawberry rhizosphere, Canada	64	28
30B	Wheat rhizosphere, Iran	17	62
R2-54-08W, R2-4-08W, R6-28-08	Wheat rhizosphere, USA	17	61
			
Pseudomonas sp. (P. fluorescens subgroup)			
R5-89-07	Wheat rhizosphere, USA	17	61
LBUM920	Spruce rhizosphere, Canada	17	Richard Hamelin
			
Pseudomonas sp. (CMR12a/CMR5c subgroup)			
CMR5c, CRM12a	Cocoyam rhizosphere, Cameroon	17	65
			
P. chlororaphis subsp. *aurantiaca* (P. chlororaphis subgroup)			
DSM19603	Unknown	17	66
CW2	Radish rhizosphere, Germany	17	67
449	Maize rhizosphere, Ukraine	17	68
464	Beet rhizosphere, Ukraine	17	69
PCM2210	Beet rhizosphere, Poland	17	PCM^*a*^
Q16	Alfalfa rhizosphere, Serbia	17	70
M12	Maize rhizosphere, Serbia	17	Dragana Josic
K27	White clover rhizosphere, Serbia	17	Dragana Josic
M71	Tomato rhizosphere, Italy	17	71
			
P. chlororaphis subsp. *aureofaciens* (P. chlororaphis subgroup)			
DSM6698^T^	River clay, The Netherlands	17	72
P2	Potato rhizosphere, Algeria	17	73
C50	Maize rhizosphere, Serbia	17	Dragana Josic
ChPhzTR18, ChPhzTR36, ChPhzTR38, ChPhzTR39	Tomato rhizosphere, France	17	49
ChPhzS23, ChPhzS24	Soil, France	17	49
66	Alfalfa rhizosphere, Uzbekistan	17	69
PA23	Soja root, Canada	74	75
O6	Soil, USA	36	76
			
P. chlororaphis subsp. chlororaphis (P. chlororaphis subgroup)			
DSM 50083^T^	Plate contaminant	17	77
			
P. chlororaphis subsp. *piscium* (P. chlororaphis subgroup)			
DSM 21509^T^	Perch intestine, Switzerland	17	78
ATCC 17411	Unknown	17	77
ATCC 17809	Unknown	17	77
ChPhzS135, ChPhzS140	Soil, France	17	49
DTR133	Tomato rhizosphere, France	17	79
SLPH10	Take-all decline soil, The Netherlands	17	16
ToZa7	Tomato rhizosphere, Greece	17	80
ChPhzTR44	Tomato rhizosphere, France	17	49
PCL1607	Avocado rhizosphere, Spain	17	81
PCL1391	Tomato root, Spain	17	35
			
P. chlororaphis (P. chlororaphis subgroup)			
Pb-St2	Sugarcane stem, Pakistan	17	82
B25	Clover rhizosphere, Serbia	17	70
30-84	Soil, USA	36	83
ATCC 17415	Soil, USA	17	77
TAMOak81	Oak, USA	17	84

a PCM, Polish Collection of Microorganisms.

### Confrontation assay.

The 63 strains of phenazine-producing Pseudomonas spp. were tested for their ability to inhibit three potato pathogens (*P*. *infestans*, V. dahliae, and *S*. *scabies*) using *in vitro* confrontation assays. For *P*. *infestans* and V. dahliae, 8.9-cm petri dishes containing 20 ml of growing medium (10% unclarified V8 agar for *P*. *infestans* and PDA for V. dahliae) were inoculated in the center with agar plugs (5 mm) taken from the margin of actively growing mycelium. Four drops (10 μl each) of 24-h-old bacterial cultures were added at the edges of the inoculated petri dishes. The plates were incubated at 20°C during 10 days for assays involving *P*. *infestans* and at 25°C during 20 days for those involving V. dahliae. The petri dishes were placed according to a randomized block design. Subsequently, the distances between the mycelium and the edge of the bacterial colonies were measured. For *S*. *scabies*, 100 μl of 6-day-old culture of *S*. *scabies* was spread to cover 8.9-cm petri dishes filled with 20 ml of OBA, and the plates were left to dry. One drop (20 μl) of phenazine-producing Pseudomonas culture was spotted in the center of the plates. After 6 days at 28°C, the inhibition zone was measured from the edge of the phenazine-producing Pseudomonas colonies to the limits of the area where *Streptomyces* vegetative growth was inhibited. For the three pathogens, values superior to 20 mm correspond to nearly complete inhibition. For each pair of potato pathogens/phenazine-producing Pseudomonas strains, four replicates were used.

### Phenazine compounds quantification in King’s B broth.

The 63 Pseudomonas strains were inoculated in 9 ml of KB broth in triplicate and incubated for 5 days at 25°C under continuous shaking. After incubation, 1 ml of bacterial culture was transferred to a 2-ml microcentrifuge tube and centrifuged for 10 min at 10,000 × *g*. The supernatant was recovered and filtered on a 0.2-μm nylon filter (Microliter Analytical Supply, Mississauga, Ontario, Canada). Standards of PCA and PCN were obtained from Ryan Scientific (Mount Pleasant, SC), and standards of 2-OH-PHZ from Angene (London, UK). Standards of PCA, PCN, and 2-OH-PHZ at a concentration of 500 ng μl^−1^ were prepared by adding 100 μl of a phenazine solution (0.01 g ml^−1^) to 9 ml of KB broth. HPLC analyses were conducted using a reverse-phase C_18_ Hydro-RP column (4 μM; 100 × 2 mm; Phenomenex, Torrance, CA). Chromatography was performed using an Agilent 1100 series HPLC system consisting of a quaternary pump, a refrigerated sample holder, and a photodiode array. The samples were maintained at 4°C, and the injection volume was 10 μl. The solvent flow rate was 750 μl min^−1^; solvent A consisted of 0.1% trifluoroacetic acid (TFA) in water, and solvent B consisted of 0.1% TFA in acetonitrile. Elution consisted of a 5-min linear gradient from 90% solvent A to 75% solvent A, followed by 4.8 min at 75% solvent A. Absorption of phenazine compounds (PCA, PCN, and 2-OH-PHZ) was measured at 254 nm, and the retention times of the phenazine standards were used to confirm the presence of phenazine compounds in the liquid cultures of the bacteria.

### Phenazine compound quantification in agar-solidified growth media.

Thirteen phenazine-producing Pseudomonas strains were selected, and confrontation assays were performed as described previously. Four agar plugs were collected from within the growth inhibition zone of each confrontation plate using a 7-mm-diameter cork borer. Care was taken to ensure plugs were taken as close as possible to Pseudomonas sp. growth. To ensure that phenazines were not produced by the plant pathogen and were not present in the growth medium prior to inoculation, plugs were also collected from plates inoculated solely with the pathogen, as well as from uninoculated plates. Plugs were then transferred to a 2-ml safe-lock microcentrifuge tube containing a single acid-washed, 5-mm-diameter stainless steel bead and ground by bead beating for 3 min at 30 Hz using a TissueLyser II (Qiagen, Venlo, The Netherlands). The resulting slurry was resuspended in 1 ml of acetonitrile by vortexing at maximum speed for 10 s, and the suspension was incubated at room temperature for 10 min with constant shaking on a tube rotisserie to allow passive diffusion of the phenazines into the organic solvent. The suspension was centrifuged at 10,000 × *g* for 5 min, and the resulting supernatant was collected and filtered through a 13-mm-diameter, 0.2-μm nylon membrane. Then, 250 μl of filtered supernatant was mixed with 750 μl of water containing 0.1% (vol/vol) formic acid. The dilution was incubated at room temperature for ∼13 h to allow insoluble components to precipitate. Then, the 1-ml dilution was filtered through a 0.2-μm nylon membrane into a 9-mm-diameter autosampler glass vial (12 × 32 mm). When appropriate, 100 μl of dilution was mixed with 900 μl of diluent (25% [vol/vol] acetonitrile and 0.075% [vol/vol] formic acid in water) prior to filtration. Phenazines were quantified by LC-MS within 2 days of the extraction.

Extracted phenazines were separated using a 1100 Series Capillary LC System (Agilent Technologies, Santa Clara, CA), equipped with a vacuum degasser, quaternary pump and autosampler. Samples were resolved at 30°C in a 100 mm × 2 mm Synergi 4 μm Hydro-RP 80 Å LC column (Phenomenex, Torrance, CA). Mobile-phase solvents comprised water (solvent A) and acetonitrile (solvent B), both supplemented with 0.1% (vol/vol) formic acid. The sample injection volume was 1 μl. Phenazines were eluted using a linear gradient increasing from 5% solvent B to 100% solvent B over the course of 8 min, followed by a steady state of 100% solvent B for 7 min. The solvent flow rate was 400 μl min^−1^. Phenazines were then detected using a time-of-flight mass spectrometer (model G6230B; Agilent Technologies) equipped with a dual electrospray ionization (ESI) ion source and operating in positive mode. A mass spectrum was acquired every second over a mass range of 100 to 1,700 *m/z*. PCA, PCN, and 2-OH-PHZ have mass-to-charge ratios of 225.058, 224.074, and 197.063 *m/z*, respectively. Their retention times were 9 to 7.0, 6.5 to 6.6, and 6.1 to 6.2 min, respectively.

Data acquisition, ion chromatogram extraction and chromatographic peak detection were carried out using the MassHunter Workstation software, version B.08.00 (Agilent Technologies). The area under the curve of each phenazine was noted and converted into concentration by comparison to the appropriate phenazine standard. Phenazine yields were ultimately normalized by medium weight. PCA (Ryan Scientific), PCN (AA Blocks, San Diego, CA), and 2-OH-PHZ (Angene) standards, as well as appropriate blanks, were included in each run. Phenazine standards comprised 800 ng ml^−1^ in an aqueous solution of 25% (vol/vol) dimethyl sulfoxide (DMSO) and 0.075% (vol/vol) formic acid, whereas blanks consisted of aqueous solutions of either 25% (vol/vol) DMSO (for standards) or 25% (vol/vol) acetonitrile (for phenazine extracts) and 0.075% (vol/vol) formic acid. Phenazine standards and DMSO blanks were centrifuged at 10,000 × *g* for 5 min, and the supernatant was transferred to an autosampler vial. Acetonitrile blanks were filtered through a 0.2-μm nylon membrane into an autosampler vial.

### *In vitro* sensitivity to phenazine compounds.

The effect of three purified phenazine compounds (PCA, PCN, and 2-OH-PHZ) on the growth of the three potato pathogens studied was evaluated under *in vitro* conditions. The three pathogens were inoculated in 8.9-cm petri dishes containing 20 ml of growth medium (V8 agar for *P*. *infestans*, OBA for *S*. *scabies*, and PDA for V. dahliae) as previously described in this work. The three phenazine compounds were dissolved and diluted in DMSO at the following final concentration: 50 μM, 10 μM, 5 μM, and 1 μM. For *P*. *infestans* and V. dahliae, two drops (50 μl each) were deposited at the edge of the petri dish. For *S*. *scabies*, one drop (50 μl) was deposited in the center of the petri dish. DMSO without phenazine compounds was used as a negative control. After 10 days (*P*. *infestans*) and 20 days (V. dahliae), the diameter of the fungal mycelium was measured. For each concentration/phenazine compound, the diameter of the fungal mycelium was compared to the negative control and expressed as a relative inhibition. For *S*. *scabies*, the diameter of the inhibition zone was measured after 6 days. The diameter of the inhibition zone was compared to the inner diameter of the petri dish and expressed as a relative inhibition. For each combination of concentration/phenazine compound/potato pathogen, three replicates were used.

### Statistical analyses.

The software R (57) and R Studio version 1.1.453 (58) was used to perform statistical analyses. The function “kruskal” from the R package “agricolae” version 1.2-8 (59) was used to perform a Kruskal-Wallis test, followed by multiple comparisons with the Bonferroni correction. Correlations between inhibition values and phenazine production were calculated using the Kendall rank correlation coefficient τ (R function cor.test [*x*, *y*, method=“kendall”]). The Wilcoxon-Mann-Whitney test was used to generate *P* values related to the associations between inhibition values and the presence or absence of phytobeneficial traits. Only traits present in five or more strains were considered.
