# Supplementary figures and images for: Inhibition of Three Potato Pathogens by Phenazine-Producing Pseudomonas spp. Is Associated with Multiple Biocontrol-Related Traits
Source: mSphere. 2021 Jun 2;6(3):e00427-21. doi: 10.1128/mSphere.00427-21 (PMC8265658; doi:10.1128/mSphere.00427-21)

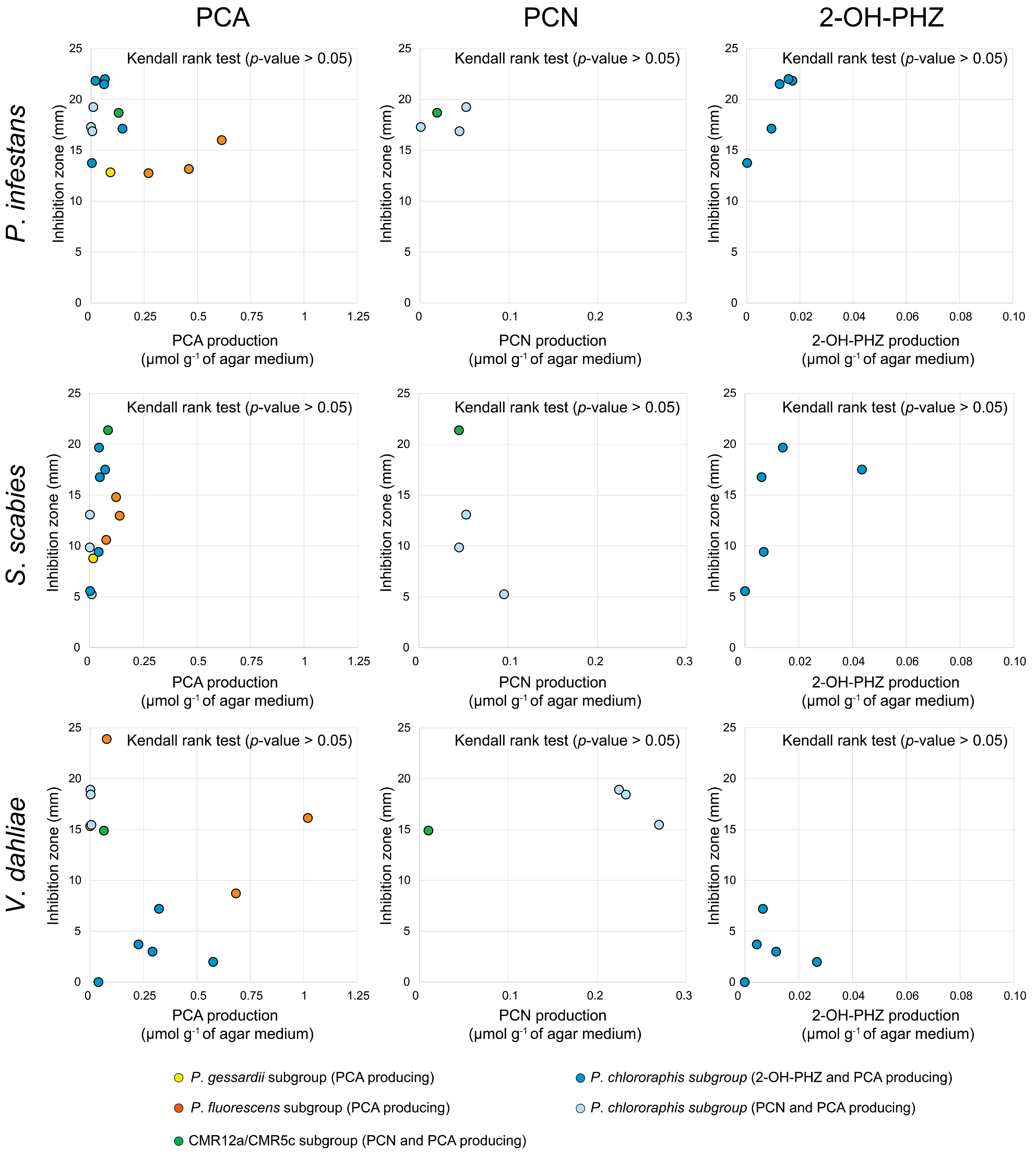

Supplement: FIG S1 [file msphere.00427-21-sf001.docx]
